# Combining affinity purification and mass spectrometry to define the network of the nuclear proteins interacting with the N-terminal region of FMRP

**DOI:** 10.3389/fmolb.2022.954087

**Published:** 2022-09-27

**Authors:** Félicie Kieffer, Fahd Hilal, Anne-Sophie Gay, Delphine Debayle, Marie Pronot, Gwénola Poupon, Iliona Lacagne, Barbara Bardoni, Stéphane Martin, Carole Gwizdek

**Affiliations:** ^1^ Université Côte d'Azur, Centre National de la Recherche Scientifique, Institut de Pharmacologie Moléculaire et Cellulaire, Valbonne, France; ^2^ Université Côte d'Azur, Institut National de la Santé Et de la Recherche Médicale, Centre National de la Recherche Scientifique, Institut de Pharmacologie Moléculaire et Cellulaire, Valbonne, France

**Keywords:** nuclear fractionation, FMRP, mRNA metabolism, nuclear protein network, proteomics

## Abstract

Fragile X-Syndrome (FXS) represents the most common inherited form of intellectual disability and the leading monogenic cause of Autism Spectrum Disorders. In most cases, this disease results from the absence of expression of the protein FMRP encoded by the *FMR1* gene (Fragile X messenger ribonucleoprotein 1). FMRP is mainly defined as a cytoplasmic RNA-binding protein regulating the local translation of thousands of target mRNAs. Interestingly, FMRP is also able to shuttle between the nucleus and the cytoplasm. However, to date, its roles in the nucleus of mammalian neurons are just emerging. To broaden our insight into the contribution of nuclear FMRP in mammalian neuronal physiology, we identified here a nuclear interactome of the protein by combining subcellular fractionation of rat forebrains with pull‐ down affinity purification and mass spectrometry analysis. By this approach, we listed 55 candidate nuclear partners. This interactome includes known nuclear FMRP-binding proteins as Adar or Rbm14 as well as several novel candidates, notably Ddx41, Poldip3, or Hnrnpa3 that we further validated by target‐specific approaches. Through our approach, we identified factors involved in different steps of mRNA biogenesis, as transcription, splicing, editing or nuclear export, revealing a potential central regulatory function of FMRP in the biogenesis of its target mRNAs. Therefore, our work considerably enlarges the nuclear proteins interaction network of FMRP in mammalian neurons and lays the basis for exciting future mechanistic studies deepening the roles of nuclear FMRP in neuronal physiology and the etiology of the FXS.

## Introduction

The Fragile X-Syndrome (FXS) represents the most common inherited form of intellectual disability and the first monogenic cause of Autism Spectrum Disorders, affecting 1/4,000 males and 1/7,000 females (Dahlhaus 2018; Maurin and Bardoni 2018; Richter and Zhao 2021). This neurodevelopmental disorder is characterized by a broad range of neurologic/psychiatric phenotypes including mental impairment, autism, attention deficit, hyperactivity, social anxiety, and epilepsy. In the majority of cases, FXS is due to the silencing of the *FMR1* gene, recently renamed as Fragile X messenger ribonucleoprotein 1, encoding FMRP (or FXP, Fragile X Protein) (Herring et al., 2022).

FMRP is an RNA binding protein, mostly cytoplasmic, able to interact with thousands of messenger RNAs (mRNAs) (Darnell et al., 2011; Ascano et al., 2012; Maurin et al., 2015; Maurin et al., 2018; Sawicka et al., 2019; Tran et al., 2019; Hale et al., 2021). These lists comprise mRNAs encoding proteins with large array of roles in cell processes, including proteins essential for the development and the function of synapses. Canonically, FMRP is defined as a translational suppressor through interactions with the translational machinery and the miRNA pathway. However, evidence exist about its capacity to enhance translation (Bechara et al., 2009; Tabet et al., 2016; Richter and Zhao 2021). It may also participate in the transport of its target mRNAs along the dendrites, within ribonucleoprotein complexes called “transport granules”, and represses their translation until they arrive at the synapses (Maurin et al., 2014; Richter and Zhao 2021). Thus, the cognitive deficiencies observed in patients with FXS are thought to result, at least in part, from the deregulation in protein translation of mRNAs bound by FMRP. In addition, FMRP may be involved in the storage and the stability of some of its mRNA targets (Davis and Broadie 2017; Richter and Zhao 2021). Lastly, FMRP directly binds different ion channels to regulate their gating, thus impacting neuronal excitability (Davis and Broadie 2017).

To accomplish its functions, FMRP interacts with numerous proteins in addition to its target mRNAs. In this context, the N-terminal domain of FMRP plays a central role (Ramos et al., 2006). Indeed, it presents a combination of Tudor and pseudo-KH patterns that promotes many of the known protein interactions of FMRP, including its homomerisation (Ramos, et al., 2006; Hu et al., 2015; Myrick et al., 2015). Intriguingly, the N-terminal domain of FMRP also contains a nuclear localization signal while its central region bears a nuclear export signal and its C-terminus two nucleolar localization sequences (Eberhart et al., 1996; Sittler et al., 1996; Bardoni et al., 1997; Taha et al., 2014), allowing the protein to enter in and exit from the nucleus and the nucleolus. Very recently, mutations within the nuclear export signal of FMRP have been found in some FXS patients, suggesting that the nucleocytoplasmic shuttling of FMRP may be important to neuronal physiology (Zeidler et al., 2021; Mangano et al., 2022). Consistently, a growing number of studies associates FMRP with nuclear functions, such as DNA damage response (Alpatov et al., 2014; Zhang et al., 2014; Chakraborty et al., 2020), certain steps in mRNA biogenesis (Didiot et al., 2009; Bhogal et al., 2011; Shamay-Ramot et al., 2015; Filippini et al., 2017; Zhou et al., 2017; Tran, et al., 2019) or their export (Kim et al., 2009; Edens et al., 2019; Hsu et al., 2019; Westmark et al., 2020; Kim et al., 2021), ribosomal RNA methylation (D'Souza et al., 2018) or nuclear pore assembly (Agote-Aran et al., 2020). However, the roles of FMRP in the nucleus of mammalian neurons remain insufficiently documented and their relevance a physiological context are still poorly understood. Notably, few information is available about the molecular mechanisms by which FMRP is involved in these different nuclear processes and whether the protein may play additional roles in this compartment. To acquire a more comprehensive representation of the functions of FMRP in the nucleus of mammalian neurons, we identified the nuclear protein partners of the N-terminal protein/protein interaction domain of FMRP in rat forebrain, using affinity pull down on nuclear fraction isolated from rat forebrain coupled to quantitative mass spectrometry analysis.

## Materials and methods

### Rat strain

Wistar rats were purchased exclusively from a commercial source (Janvier, St Berthevin, France). All animals were handled and treated in accordance with the ARRIVE Guidelines. Animals had free access to water and food. Lightning was controlled as a 12 h light and dark cycle and the temperature maintained at 23°C ± 1°C. The protocols for PND 14 pups euthanasia by decapitation and the preparation of primary neuronal cultures from rat embryos at E17 were approved by the Animal Care and Ethics Committee (APAFIS #18647-2019011110552947v3). For biochemical analyses, forebrains were immediately excised, frozen in liquid nitrogen and stored at −80°C until use.

### Nuclear preparation

Forebrains of PND 14 rats were homogenized in ice-cold hypotonic buffer at 1.5 mM MgCl2 (see composition of all buffers in Supplementary Material) using a glass-Teflon homogenizer. Igepal (MP) was added at a final concentration of 0.3% and the homogenate was filtrated on nylon Cell Stainer 70 μm (Falcon) (Total fraction). The filtrate was centrifuged at 800 g for 5 min at 4°C. The supernatant was removed (Cytoplasmic fraction). After resuspension of the pellet in hypotonic buffer at 0.5 mM MgCl2 and filtration, 1.1 volume of Optiprep (StemCell) was added. After a gentle homogenization, the mix was subjected to centrifugation at 5,000 g for 15 min at 4°C. The pellet, corresponding to the purified nuclei, was resuspended in native lysis buffer, sonicated 7 times at 15% of the power (Sonic ruptor 400, Omni International) and centrifuge at 4,000 g for 15 min at 4°C (Nuclear fraction). Protein concentration was determined using standard Bradford assay. Buffer compositions are available in the Supplementary Material.

### Glutathione S-transferase-pull down

pGEX-4T1 plasmids encoding the Glutathione S-transferase (GST) or the N-terminal fragment (amino acids 1–213) of human FMRP fused to the GST (GST-FNT) were transfected to in *E. coli* BL21 (DE3) bacteria (Invitrogen) and recombinant GST or GST-FNT were produced and purified as previously described (Khayachi et al., 2018). 50 μg of GST or 100 μg of GST-FNT purified recombinant proteins were incubated 1 h at 4°C under soft rotation with 25 μl of Glutathione Sepharose 4B beads (GE HealthCare). Beads were washed twice with PBS and bound recombinant proteins were cross-linked to the beads using 30 mM dimethyl pimelimidate (Sigma) according to the previously published protocol (Pronot et al., 2021). 6 mg of proteins from nuclear lysates were incubated with 25 μl of GST-FNT or GST cross-linked beads overnight at 4°C under soft rotation. Beads were washed three times for 5 min at 4°C in wash buffer. To decrease unspecific or indirect bindings, the beads were further washed in high stringency wash buffer containing 500 mM NaCl. Proteins bound to the beads were then eluted in 30 μl of Laemmli buffer for 10 min at 95°C. Buffer compositions are available in the Supplementary Material.

### Mass spectrometry analysis

After separation by short SDS-PAGE, gel slicing in two bands per lane and in gel Trypsin digestion, proteins from PND14 rat forebrain nuclear extract or isolated from GST and GST-FNT pull down were identified by liquid nano-chromatography coupled to tandem mass spectrometry as described in the Supplementary Material and Methods.

### Bioinformatics

Uniprot protein identifiers were converted into Entrez Gene identifiers using the Uniprot or the db2db conversion tools. When rat datasets were compared to human datasets, homologs of our dataset were identified using DIOPT or Blast. Enrichment analysis for Gene Ontology terms or REACTOME pathways against the Rattus Norvegicus Proteome and annotations for Uniprot Keywords were performed using DAVID. Network analysis were conducted using STRING (v11.5) with physical subnetwork mode (excluding Text mining sources) and a medium confidence score. Clusters prediction and annotation were performed using Cytoscape (v3.8.0) implemented by the StringApp.

### Proximity ligation assay on primary neuronal culture

Hippocampal neurons were prepared as previously described (Schorova et al., 2019). The Duo-link^®^ using PLA Technology kit (Sigma-Aldrich) was used for the proximity ligation assay, accordingly to the manufacturer instructions. The primary antibodies incubation was performed overnight at 4°C as indicated in the Supplementary Material. Neuronal cells in the cultures were identified upon their MAP2 labelling. Confocal images were acquired with a ×63 oil immersion lens (numerical aperture NA 1.4) on an inverted TCS-SP5 confocal microscope (Leica Microsystems, Nanterre, France).

## Results

At the steady state, FMRP is mainly cytoplasmic and barely detectable in the nucleus. Indeed, nuclear accumulation of FMRP was essentially detected in cell lines exogenously over-expressing full length, mutated or truncated proteins (Eberhart, et al., 1996; Fridell et al., 1996; Sittler, et al., 1996; Willemsen et al., 1996; Bardoni, et al., 1997; Feng et al., 1997; Tamanini et al., 1999a; Taha, et al., 2014). It has been estimated from subcellular fractionation of human lymphoblastoid cells that approximatively 2%–4% of the endogenous FMRP are present in the nuclear compartment (Feng, et al., 1997). In mammalian neurons, only immuno-electron microscopy or PLA approaches provided sufficient sensitivity to detect endogenous FMRP-labeled particles in the nucleus (Feng, et al., 1997; Bakker et al., 2000; Filippini, et al., 2017), suggesting that only a small proportion of the protein goes to the nucleus and/or that its passage is very transient. In this context, the identification of the nuclear protein interactome of FMRP appears particularly challenging. To overcome this limitation, we chose to use a GST pull-down co-purification approach on an enriched nuclear fraction from rat forebrain followed by mass spectrometry analysis.

### Preparation of an enriched nuclear fraction from developing rat brain

**FIGURE 1:**
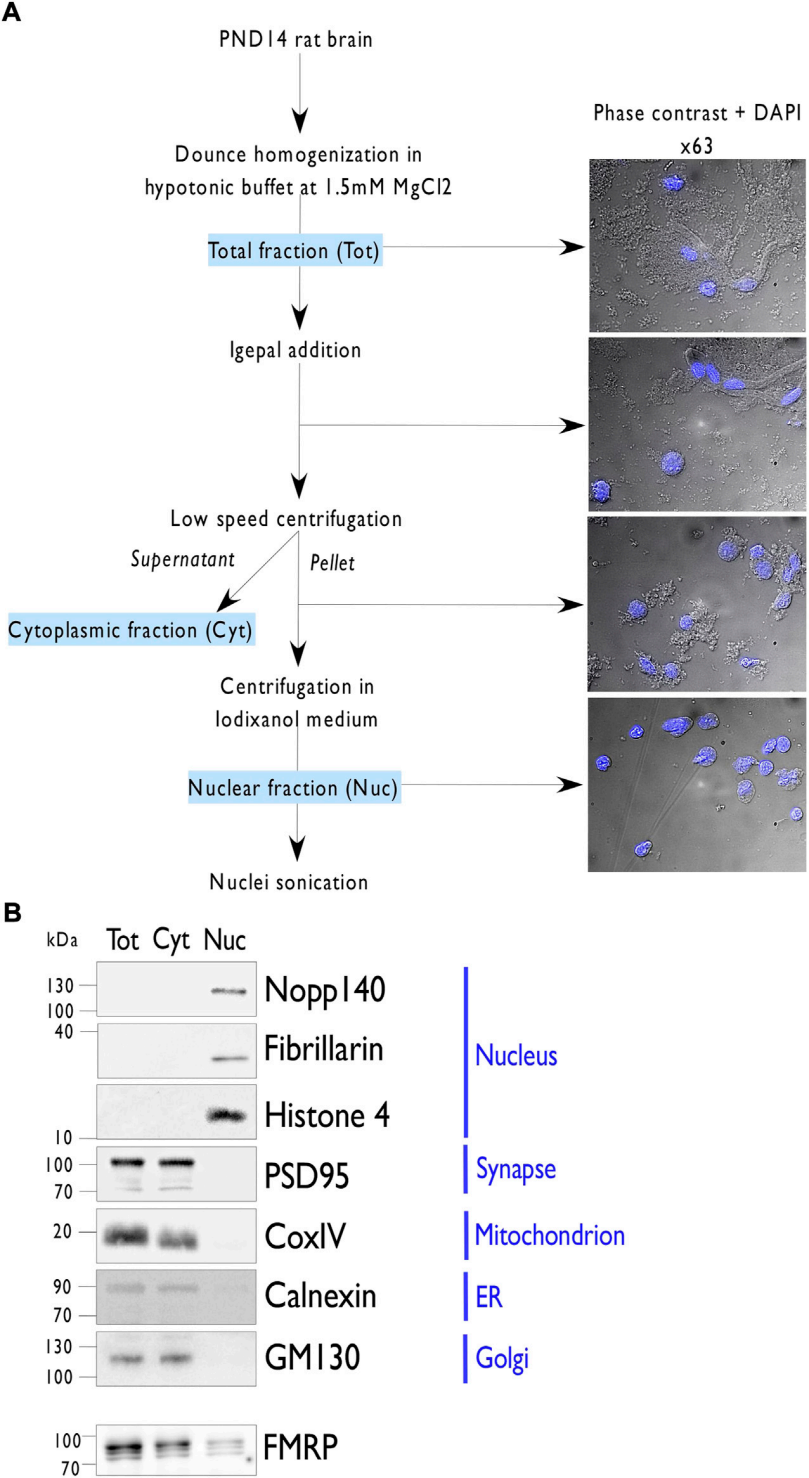
Nuclei isolation from PND14 rat forebrains. **(A)**. Representation of the protocol used to purify nuclei from postnatal day 14 (PND) rat forebrains. Briefly, forebrains were dounced in hypotonic buffer and then supplemented with 0.3% of Igepal detergent. After filtration, the total forebrain lysate (Tot) was subjected to low-speed centrifugation to separate the nuclei in the pellet from the cytoplasmic material in the supernatant (Cyt). After resuspension of the pellet in hypotonic buffer, 1.1 volume of Optiprep was added. After gentle homogenization, the mix was subjected to high-speed centrifugation and the purified nuclei (Nuc) were recovered in the pellet. To extract nuclear proteins, purified nuclei were resuspended in a native lysis buffer, sonicated, and clarified by high-speed centrifugation. To follow the purity fraction, images are acquired by phase contrast and superposition with DAPI staining at the indicated steps. **(B)**. Immunoblot analysis of 10 µg of proteins from the indicated fractions (Tot, Cyt, Nuc) per lane and detected by western blotting using antibodies against different subcellular markers or FMRP.

In both human and rodent, FMRP, essential for proper neuronal development, is highly expressed in neonatal brain and declines to reach low levels of expression in adults (Bonaccorso et al., 2015; Prieto et al., 2021). In this context, postnatal day (PND) 14 represents an interesting period to identify the nuclear interactome of FMRP in rat as it combines intense synaptogenesis (Semple et al., 2013) and high levels of the protein (Bonaccorso, et al., 2015). To prepare the nuclear protein fraction, whole nuclei were isolated from the forebrain of PND14 rats *via* a series of differential centrifugations (Figure 1). The quality of the fractionation was verified by phase contrast microscopy (Figure 1A) as well as by western blotting (Figure 1B; Supplementary Figure S1). As observed by light microscopy, each step of the workflow increased the purity of the preparation, until a fraction highly enriched in pure and intact nuclei is obtained (Figure 1A). To address the relative purity of the nuclear extract, protein samples from the total (Tot), cytoplasmic (Cyt), and nuclear (Nuc) lysates were analyzed by western blotting for specific markers of various sub-cellular compartments (Figure 1B; Supplementary Figure S1). The nuclear fraction results to be highly enriched in the nuclear markers Histone H4, Fibrillarin, and Nopp140 compared to the other fractions and devoid of markers for synapses (Synaptotagmin), mitochondrion (CoxIV), endoplasmic reticulum (Calnexin), and Golgi apparatus (GM130). Interestingly, FMRP is also found in the nuclear fraction (Figure 1B). We estimated that around 2% of the endogenous protein is localized in the nucleus, comparable to what has been previously described for human lymphoiblastoid cells (Feng, et al., 1997).

**FIGURE 2:**
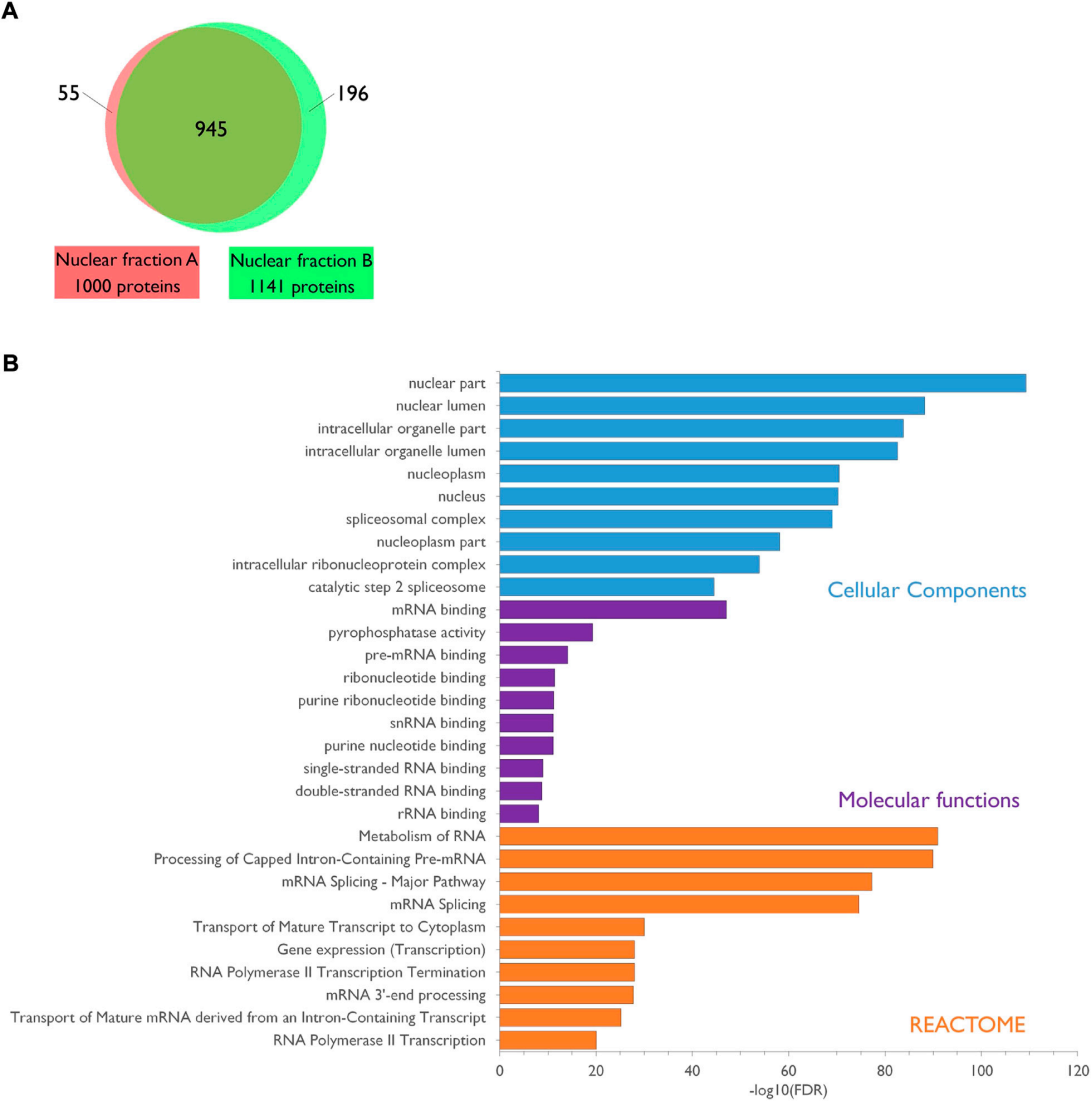
Characterization of the PND14 rat forebrain nuclear proteome. **(A)**. Venn diagram showing the overlap between the two protein sets identified by mass spectrometry analysis of two independent preparations of nuclear extracts from PND14 rat forebrains. Details are available in Supplementary Table S1. **(B)**. The nuclear proteome dataset was subjected to enrichment analysis for GO Cellular Components, GO Molecular Functions and Reactome pathways against the rat proteome. Categories were classified according to the–Log10(FDR). Details are available in Supplementary Table S2.

Lastly, to evaluate the quality of the purified fraction, two independent nuclear lysates from PND14 rat forebrains were subjected to proteomics analysis after protein separation by short SDS-PAGE, gel slicing in two bands per lane and in gel Trypsin digestion. A total of 1,196 distinct proteins was identified and 945 of them (79%) were present in both sets of nuclear preparation (Figure 2A; Supplementary Table S1). Gene Ontology (GO) enrichment analysis showed that the top 10 for enriched GO Cellular Components terms is clearly associated to the nuclear compartment whereas the top 10 for enriched GO Molecular Function terms refers essentially to RNA binding activities. Consistently, the top 10 for enriched GO Biological Processes or REACTOME pathways revealed the involvement of the identified proteins in different steps of RNA metabolic processes (Figure 2B; Supplementary Table S2). Altogether, these data confirm the enrichment of the samples in nuclear components thus highlighting the quality of the nuclei preparation.

### Identification of a nuclear interactome of FMRP by pull-down purification and mass spectrometry analysis

**FIGURE 3:**
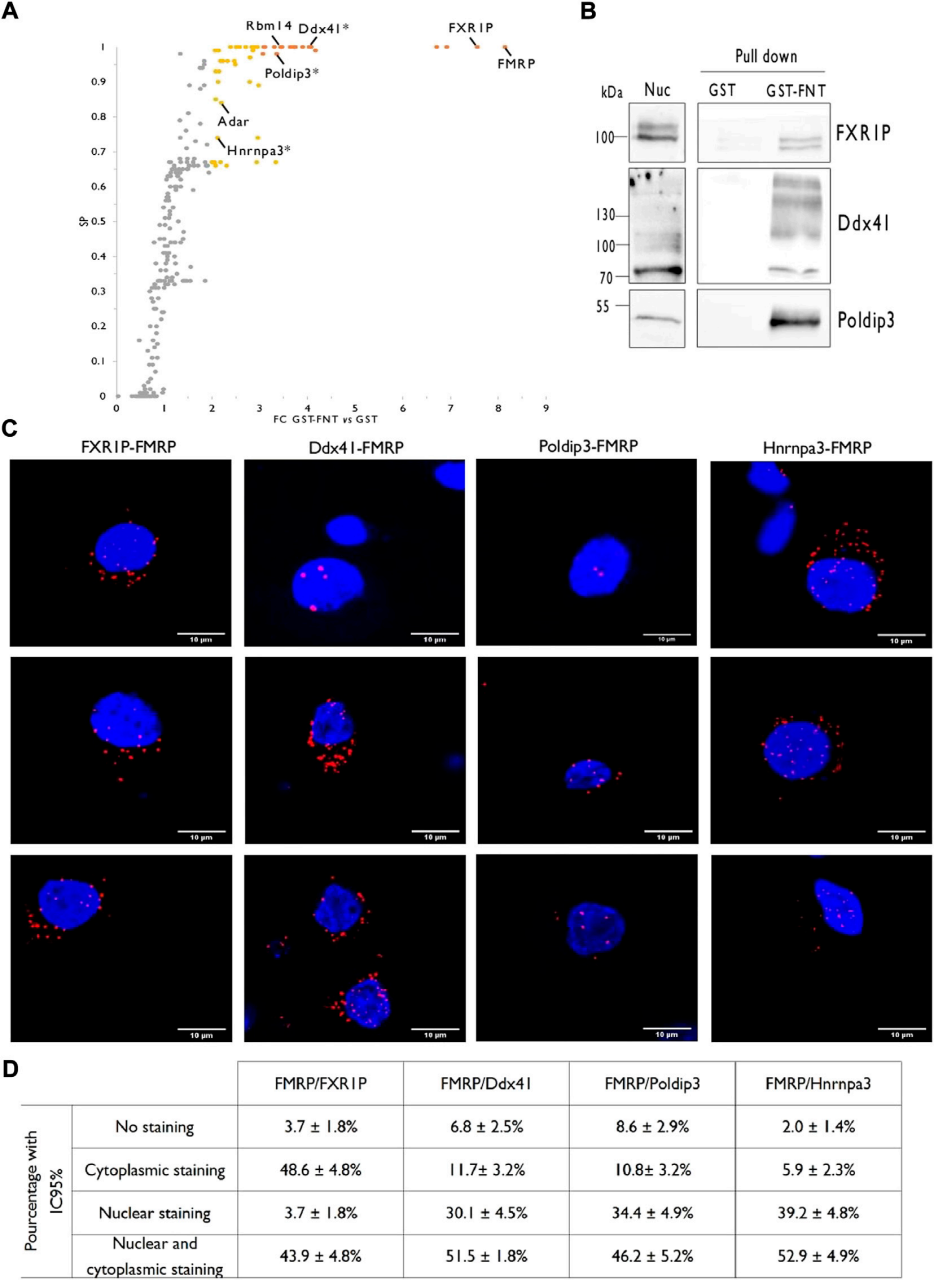
Identification of rat forebrain nuclear FMRP-interacting proteins. **(A)**. Volcano plot highlighting proteins differentially co-purified in triplicate pull-down experiments using the GST-FNT recombinant protein versus the GST alone. Statistically significant differences were assessed using the Saint-Express tool. Unenriched proteins are displayed in gray, while enriched proteins presenting a FC > 3.00 and SP > 0.7 are displayed in orange, and were classified as “high-confidence” interactor. Yellow dots corresponding to all other significant proteins with FC > 2.0 and SP > 0.5, and were referred as “medium-confidence” interactor. Know and novel (*) FMRP interacting proteins are noted in the scatter plot. **(B)**. Immunoblot analysis of identified FMRP-interacting proteins. Pull down experiments were conducted with GST-FNT and GST (negative control) immobilized on Glutathione Sepharose with nuclear fractions. Protein retained on the beads were resolved by SDS-PAGE and processed for western blotting using antibodies against FXR1P, Poldip3 or Ddx41. **(C)**. Representative confocal images of the interaction between endogenous FXR1P, Ddx41, Poldip3 or Hnrnpa3, and FMRP detected by Proximity Ligation Assay (PLA) in rat primary hippocampal neuron cultures. Neuronal cells were identified upon their MAP2 labelling (not shown). **(D)**. Percentage of neurons presenting no PLA dots (No staining), PLA dots exclusively in the cytoplasm (Cytoplasmic staining), exclusively in the nucleus (Nuclear staining) or in both the cytoplasm, and the nucleus (Cytoplasmic and nuclear staining) for the indicated interactions. The percentages with the 95% Confidence Interval were calculated from 107 (FMRP/FXR1P), 103 (FMRP/Ddx41), 93 (FMRP/Poldip3), and 103 (FMRP/Hnrnpa3) neurons in culture processed from three independent experiments.

The nuclear lysates from PND14 rat forebrains were used to investigate the nuclear interactors of FMRP by stringent GST pull-down assays using the recombinant N-terminal protein/protein interaction domain of FMRP fused to the GST (GST-FNT) as a bait (Supplementary Figure S2), or the GST alone as negative control, as detailed in the Material and Methods section. Three independent GST-FNT co-purifications with their respective GST controls were analyzed by liquid chromatography coupled to tandem mass spectrometry (LC-MS/MS). MS data were processed as described in the Material and Methods section and the differential statistical analysis for each identified prey was performed using SAINTexpress, the upgraded implementation of the Significance Analysis of INTeractome Tool (Mellacheruvu et al., 2013; Teo et al., 2014). As previously reported (Guard et al., 2019), proteins presenting a fold-change enrichment (FC) cutoff of >3.00 and a score probability (SP) cutoff >0.7 were classified as “high-confidence” interactor whereas we referred to all other proteins with a FC cutoff of >2.00 and a SP cutoff >0.5 as “medium-confidence” interactors. This workflow led to a list of 55 FMRP-interacting nuclear candidates, with 20 proteins satisfying the “high-confidence” requirements and 35 passing the “medium-confidence” standards (Figure 3A; Supplementary Table S3). Noteworthy, our dataset presents five reported FMRP partners previously identified by different approaches: FMRP itself (Ramos, et al., 2006; Hu, et al., 2015; Myrick, et al., 2015), FXR1P (Fragile X Related Protein 1) (Zhang et al., 1995), the zinc finger RNA-binding protein ZFR (Worringer et al., 2009), the splicing factor Rbm14 (Zhou, et al., 2017), and the mRNA editing enzyme Adar (Shamay-Ramot, et al., 2015; Filippini, et al., 2017). Besides, two proteomic screenings by affinity pull-down with the N-terminal domain of FMRP as bait were previously conducted using total cell extracts from HEK293 cells as source of preys (He and Ge 2017; Taha et al., 2021). The comparison of our list with these dataset brought out five proteins in common: FMRP, FXR1P, the chromatin-remodeling factor CHD4 and the transcriptional factors TCF20 and ZNF638.

### Validation of novel nuclear FMRP interacting proteins

To go further with the validation of the proteomic screen, we then verified the interaction between FMRP and FXR1P or three novel candidates, Ddx41, Poldip3, and Hnrnpa3 by target specific approaches.

As FMRP, FXR1P is a predominantly cytoplasmic RNA binding protein, playing a role in the local translation or the stability of certain mRNAs (Khlghatyan and Beaulieu 2018; George et al., 2021). Interestingly, FXR1P forms heterodimers with FMRP and these proteins have common mRNA targets (Tamanini et al., 1999b; Darnell et al., 2009). FXR1P is also able to shuttle between the nucleus and the cytoplasm (Tamanini et al., 1999a; Bakker, et al., 2000). Tran et al. recently showed that both FMRP and FXR1P interacts with ADAR1 in HeLa cells to positively and negatively regulate A to I RNA editing, respectively. They proposed that the proteins may contribute to the alterations of RNA editing that they observed in the post-mortem brain of ASD patients (Tran, et al., 2019). In cancer cells, FXR1P was involved in the recruitment of transcription factors to gene promotors (Fan et al., 2017). We first performed GST-FNT pull down experiments on nuclear fraction from PND14 rat forebrain followed by FXR1P detection by western blotting and found a specific binding of the protein with GST-FNT while it was barely detected in the GST control lane (Figure 3B). Next, to assess the interaction of FXR1P with endogenous FMRP in the nucleus of neurons, we conducted Proximity Ligation Assays (PLA) in cultured hippocampal rat neurons at 21 days *in vitro* (DIV). Consistent with the known interaction between both proteins in the cytoplasm, the PLA for FMRP and FXR1P showed many cytoplasmic dots in neurons (Figures 3C,D). No PLA dots were detected as background in the absence of primary antibodies (Supplementary Figure S3). Besides, some dots were also detected in the nucleus, thus confirming the association of the two proteins in the nuclear compartment of neurons. It is very interesting to underline that the profile of the PLA labelling is heterogeneous, with almost 44% of the neurons presenting dots in both the nucleus and the cytoplasm, 4% in the nucleus only and about 48% in the cytoplasm only (Figure 3D). This diversity suggests that the interaction between FMRP and FXR1P in the nucleus may depend on the neuronal cell types or their levels of activity for example.

Ddx41 is a multi-functional DEAD box helicase both nuclear and cytoplasmic. It is involved in pre-mRNA splicing and counteracts the accumulation of R-loops in promoter regions of active genes thus participating in genome stability. The protein also plays a role as a cytosolic DNA-sensor in DNA-mediated innate immunity (Andreou 2021; Mosler et al., 2021). GST-FNT pull down on nuclear fractions from rat forebrain followed by western blotting showed a specific binding between the N-terminal domain of FMRP and Ddx41 (Figure 3B) whereas PLA reveals an interaction between the endogenous proteins in the nucleus and the cytoplasm of neurons (Figures 3C,D). Nonetheless, as in the case for the FMRP-FXR1P interaction, the profile of the labelling is heterogeneous (Figure 3D) suggesting that the association Ddx41-FMRP may depends on the neuronal status.

Poldip3 (Polymerase δ-interacting protein 3), also called PDIP46 (46 kDa Polymerase δ -interacting protein) or SKAR (S6K1 Aly/REF-like target) is distributed in both the nucleus and the cytoplasm. Poldip3 is involved in cellular DNA replication and genome stability (Wang et al., 2016; Bjorkman et al., 2020). In addition, the protein is a member of the exon-exon junction complex and recruits the SK6 kinase on newly synthesized mRNA, which will later enhance the pioneer round of translation of spliced mRNA (Ma et al., 2008). Lastly, by interacting with the TREX complex, Poldip3 would participate in the nuclear export of mRNA (Folco et al., 2012). As illustrated Figures 3B,C, an interaction between FMRP and Poldip3 is detected by pull down assay and PLA. Images from PLA show an association of the endogenous proteins either in the nucleus or in both the nucleus and the cytoplasm of neurons (Figures 3C,D), with pattern consisting in 2–4 dots in the nucleus and also few spots in the cytoplasm (Figure 3C).

Lastly, we analyzed by PLA the interaction of FMRP with a third novel interactor, Hnrnpa3 (Heterogeneous nuclear ribonucleoprotein A3). In the nucleus, Hnrnpa3 recognizes single-stranded telomeric DNA and is involved in telomere maintenance (Huang et al., 2010). Besides, this protein is also implicated in the splicing, the stability and the cytoplasmic trafficking of mRNAs (Ma et al., 2002; Papadopoulou et al., 2012; Kwon et al., 2021). We showed here that consistent with the pull down screen, endogenous FMRP and Hnrnpa3 interact in neurons, mainly in the nucleus only or in both the nucleus and the cytoplasm (Figures 3C,D).

Altogether, these target specific analyses further validated the pull down approach on nuclear-enriched fractions from PND14 rat forebrains to identify a nuclear interactome of FMRP.

### Functional categorization of the nuclear interactome of FMRP

**FIGURE 4:**
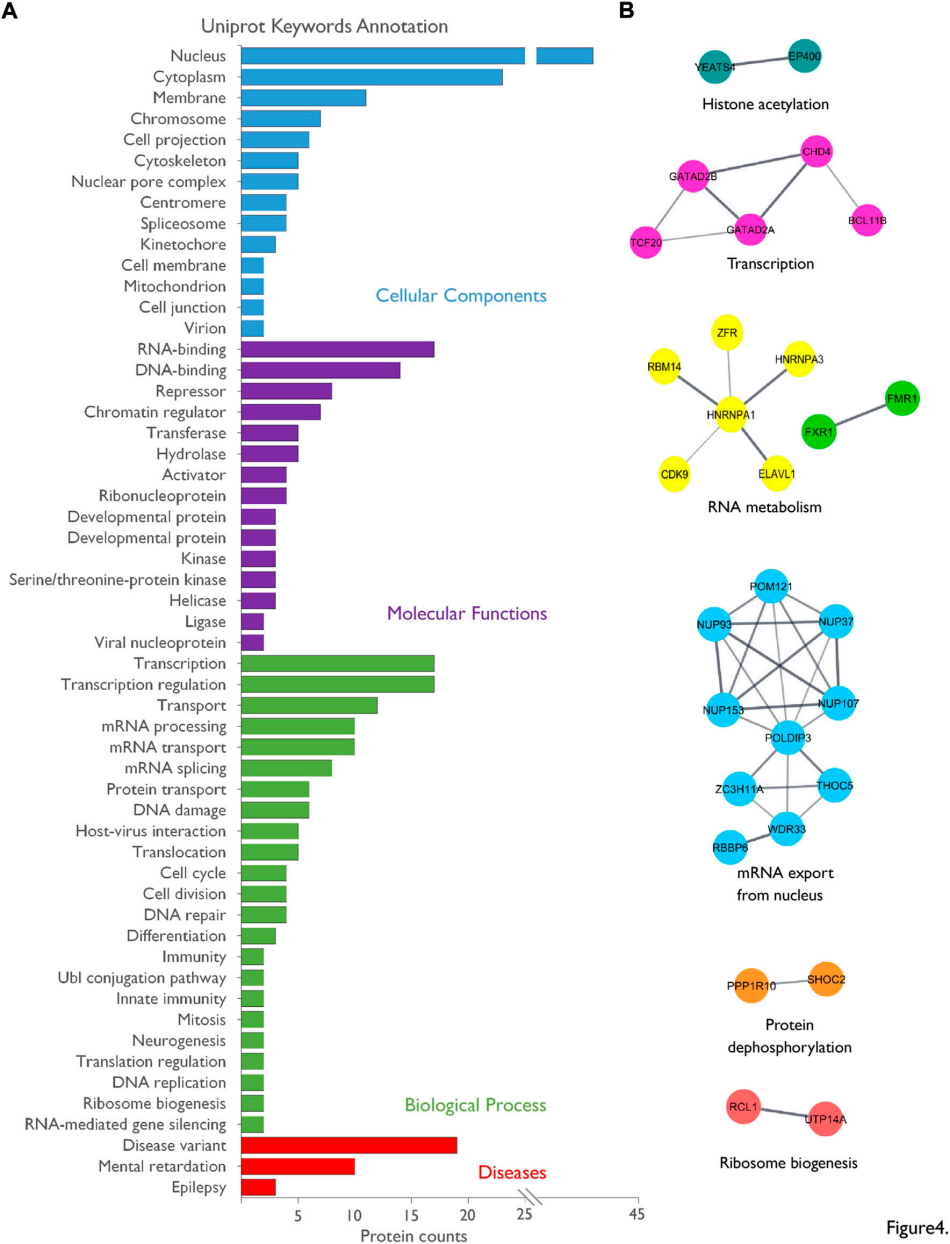
Bio-informatics analysis of the nuclear interactome of FMRP. **(A)**. The list of the nuclear FMRP-interacting proteins was subjected to Uniprot Keywords (UP-KW) annotation analysis using the reviewed human homologues as data source: UP-KW for Cellular Components, UP-KW for Molecular functions, UP-KW for Biological process, and UP-KW for Diseases. Terms were ranked according to the number of proteins in the category. Details are available in Supplementary Table S4. **(B)** A network was built on physical interaction to complexes predicted by STRING using all identified FMRP-interacting proteins candidates as source, with an interaction confidence of 0.4 or greater and based on databases and experiments sources. The STRING network was imported into the Cytoscape application and clusters were created and annotated through the StringApp plug-in.

We identified 55 FMRP-interacting protein candidates. To assess the biological meaning of this nuclear interactome, we performed annotation analyses for UniProt Key Words (UP-KW) on the reviewed human homologues of our rat dataset (Figure 4A; Supplementary Table S4). Consistent with our strategy based on affinity purification on nuclear enriched extracts, 47 proteins are associated with the UP-KW for Cellular Component “nucleus”, i.e., 85% of the list, with 17 proteins (30%) presenting both a nuclear and a cytoplasmic localization (Supplementary Table S4). Moreover, “RNA-binding”, “DNA-binding,” and “Chromatin regulator” appeared among the highest UP-KW for Molecular Functions. The annotation for UP-KW linked to Biological Processes highlighted among the most representative terms the involvement of the FMRP partners in several steps of mRNA biogenesis as “transcription”, “mRNA splicing” or “transport”, besides to “DNA damage”. Consistently, a network analysis based on protein association to physical complexes revealed clusters associated to histone acetylation, transcriptional regulation, mRNA metabolism, mRNA export, translational regulation as well as protein de-phosphorylation and ribosome biogenesis (Figure 4B). Lastly, UP-KW annotation for Diseases respectively associated 10 and 3 proteins to the “Mental retardation” and “Epilepsy” categories, which fully correlates with phenotypes associated with the FXS (Figure 4A).

## Discussion

While, in the past, the nuclear presence of FMRP was mainly associated with the its role in RNA export from nucleus to cytoplasm, now increasing evidence strongly suggest the implication of FMRP in nuclear processes. However, the molecular mechanisms underlying these nuclear functions notably in neurons are currently missing. In the present study, we identified 55 protein candidates interacting with of the N-terminal domain of FMRP from nuclear PND14 rat forebrain extracts. A bibliographic analysis revealed that five proteins identified in this dataset, FMRP itself (Ramos, et al., 2006; Hu, et al., 2015; Myrick, et al., 2015), FXR1P (Fragile X Related Protein 1) (Zhang, et al., 1995), the zinc finger RNA-binding protein ZFR (Worringer, et al., 2009), the splicing factor Rbm14 (Zhou, et al., 2017), and the mRNA editing enzyme Adar (Shamay-Ramot, et al., 2015; Filippini, et al., 2017), were previously reported to associate with FMRP in target specific studies. Besides, comparing our dataset with two published proteomic screenings using affinity pull-down with the N-terminal domain of FMRP revealed five proteins in common: FMRP, FXR1P, the chromatin-remodeling factor CHD4 and the transcriptional factors TCF20 and ZNF638. This narrow overlap may largely be explained by distinct experimental conditions, notably the use of HEK 293 cell line extracts and/or the absence of a nuclear enrichment in the earlier proteomic approaches (He and Ge 2017; Taha, et al., 2021). Besides, we could not find the Nuclear FMRP Interacting Protein 1 (NuFIP1), known to interact with N-terminal domain of FMRP (Bardoni et al., 1999), neither in the list of candidate nuclear partners, nor in the nuclear proteome. Yet, NuFIP1 is a nucleocytoplasmic shuttling protein (Bardoni et al., 2003) and its expression in time and space in the brain is not known. Its absence in our datasets may be explain by a low level of expression and/or a predominantly cytoplasmic localization in the PND14 rat forebrain. In the future, it will be very informative to complement the present results with pull-down experiments performed on nuclear extracts prepared from the forebrain or brain sub-structures of rats of different ages, in order to access the variation of the FMRP nuclear interactome across brain regions and over the life span.

In the present study, we found that FXR1P, known to interact with FMRP in the cytoplasm, also binds the protein in the nucleus. In addition, we experimentally validated three novel interactors, Ddx41, Poldip3, and Hnrnpa3. It should be noted here the identification of protein–protein interactions by pull down affinity purification may, as co-immunoprecipitation, result from direct physical interactions but also from indirect interactions. Besides, in the case of GST-pull down experiments, unspecific binding may result from the association of some proteins to the beads or with the GST tag. To identify and discard these background proteins, we used a pull down condition with GST alone cross-linked to the glutathione beads as negative control. In addition, to minimize indirect binding, we introduced a washing step with 500 mM NaCl prior to elution. However, some of the interactions we detected may still arise from indirect protein/protein or protein/nucleic acid binding with direct and specific preys. Nonetheless, whether FMRP interacts directly with the identified candidates or in the context of ribonucleoprotein complexes, our dataset clearly reflects an association of FMRP with nuclear machineries, notably with those involved in pre-mRNAs biogenesis. Moreover, our data indicate that the profile of the endogenous interaction between FMRP and its nuclear partners in neurons may vary, likely reflecting differences in cell characteristics such as neuronal cell type or activity.

The identification of the biological pathways associated with our dataset as well as the network analysis match with the nuclear functions reported to be modulated by FMRP, including mRNA editing (Bhogal, et al., 2011; Shamay-Ramot, et al., 2015; Filippini, et al., 2017; Tran, et al., 2019), mRNA nuclear export (Kim, et al., 2009; Edens, et al., 2019; Hsu, et al., 2019; Westmark, et al., 2020; Kim, et al., 2021) or DNA damage response (Alpatov, et al., 2014; Zhang, et al., 2014; Chakraborty, et al., 2020). Our work thus provides valuable insights and a foundation for future mechanistic investigations of the overlooked nuclear functions of FMRP in neurons. Interestingly, we detected an interaction in the nucleus of neurons between FMRP and FXR1P, which positively and negatively regulate ADAR-mediated RNA editing, respectively. Whether and how this interaction may modulate the modification of their respective or common RNA targets will need to be further explored.

Transcription and splicing recently emerged as a key nuclear process regulated by FMRP. Indeed, large-scale screens identified splice site variations between mouse or drosophila models of FXS and their wild-type controls (Brooks et al., 2015; Shah et al., 2020). It has been proposed that these alterations result from the dysregulated translation of FMRP target mRNAs encoding chromatin modifying enzymes (Shah, et al., 2020; Hale, et al., 2021) that would alter the profile of histones post-translational modifications and in turn, mRNA splicing (Shah, et al., 2020). Similarly, other epigenetic changes would affect transcriptional activation (Korb et al., 2017). Interestingly, the current dataset presents several factors involved in chromatin remodeling, transcriptional regulation or mRNA splicing. Consistent with these results, Kim, et al. (2009) detected an association between FMRP and nascent transcripts on lampbrush chromosomes in amphibian oocytes. In another context, Alpatov and colleagues showed in mouse embryonic fibroblasts an association of FMRP with chromatin fraction, enhanced by stress replication (Alpatov, et al., 2014). Our list includes the reported FMRP-interacting protein Rbm14 (Zhou, et al., 2017), the helicase Ddx41 (Figure 3) and Hnrnpa3 (Figure 3), all connected to mRNA splicing. These results are in agreement with studies describing a direct role of FMRP in the splicing of some mRNAs including its own mRNA (Didiot et al., 2008; Zhou, et al., 2017) and complement to the indirect effects outlined above. In the same way, FMRP could directly participate in gene transcription and/or mRNA export, for example through interaction with Poldip3 (Figure 3).

Many studies have shown that the processes involved in mRNA metabolism, from transcription to nuclear export and cytoplasmic trafficking, are tightly coupled (Vitaliano-Prunier et al., 2012; Bjork and Wieslander 2017; Woodward et al., 2017; Garland and Jensen 2020). This functional coordination results from physical interactions between members the different mRNA biogenesis machineries, which synchronize or cross-stimulate the connected processes. In this line of view, FMRP may act as a hub protein that could follow, partially or completely, the fate of its target mRNAs. Interestingly, some studies indicate that other neuronal mRNA-binding protein such as ELAV or SMN proteins, involved in mRNA dendritic transport in neurons, transit through the nucleus and participate in early post-transcriptional regulatory events such as pre-mRNA splicing or poly-adenylation (Colombrita et al., 2013; Raimer et al., 2017; Ravanidis et al., 2018; Jung and Lee 2021; Wei and Lai 2022). We propose here that the workflow we set up, combining an efficient nuclear fractionation from rat forebrain coupled to affinity pull down followed by identification using tandem mass spectrometry, could be adapted to assess the nuclear interactome of these dual-distributed but predominantly cytoplasmic neuronal factors.

Lastly, annotation analysis indicates that twenty-one of the FMRP-interacting proteins identified in this work present one or more genetic variants involved in a disease and/or are connected to “mental retardation” or “epilepsy”. As an example, identification of *de novo* mutations in patients linked the FMRP partner candidates Tbl1xr1 and Chd4 to autism spectrum disorders (Coe et al., 2019; O'Roak et al., 2012; Quan et al., 2020). Strikingly, about 50% of the FXS males and 20% of the FXS females meet the criteria for ASD (Kaufmann et al., 2017). More generally, several studies have highlighted links between RNA metabolism and neurodevelopmental and neurological diseases (Chatterjee et al., 2021), with shared molecular pathways between various disorders. We believe that our proteomic screen, clearly associating FMRP with mRNA biogenesis, may provide leads to further explore these molecular links.

## Data Availability

The mass spectrometry proteomics data presented in this study have been deposited to the ProteomeXchange Consortium *via* the PRIDE partner repository (https://www.ebi.ac.uk/pride/archive/) with the dataset identifier PXD034157.

## Supplementary material

Supplementary Figure S1 Detection of Nopp140, Fibrillarin, and Histone H4 in the total lysate from PND14 rat forebrain. To appreciate the levels of the nuclear markers Nopp140, Fibrillarin, and Histone H4 in the total lysate of PND14 rat forebrain compared to the nuclear fraction, 10, 20, and 50 µg of proteins from the total lysate (Tot) or 10 µg of proteins from the nuclear fraction (Nuc) were subjected to immunoblotting using the indicated antibodies and then the appropriate horseradish peroxidase (HRP)-conjugated secondary antibodies. Proteins were visualized using the Enhanced chemiluminescence western blot kit from Millipore (ECL) or the SuperSignal West Femto Maximum Sensitivity substrate from Thermo Scientific (Ultra sensitive ECL) at the indicated time of acquisition. Our results indicate that the visualization of the nuclear markers in the total fraction compared to their detection in the nuclear lysate requires higher amount of proteins, associated with longer time of exposure and/or ultra sensitive ECL substrate, thus reflecting a clear enrichment of the nuclear fraction in nuclear proteins.

Supplementary Figure S2 Description of the GST-FNT bait. High panel: Schematic diagram of the human N-terminal fragment (1-213aa) fused to the GST tag used in this study (GST-FNT). Major domains (Ag1, Ag2, KH0) and the nuclear localization signal (NLS) of FMRP are highlighted. Low panel: Coomassie stained gel of the purified GST and GST-FNT recombinant proteins. Black arrow indicates GST and red arrow indicates GST-FNT.

Supplementary Figure S3 Negative control for Proximity Ligation Assays (PLA). Representative confocal images of primary hippocampal neurons in culture processed with the PLA Duo-link® kit (Sigma-Aldrich) without primary antibodies (Scale bar = 50 μm). Enlargement of delimited area is also shown (Scale bar = 10 μm). No PLA dots could be detected as background signal.

Supplementary Table 1 PND14 rat forebrain nuclear proteome. 10 μg of proteins from two independent nuclear preparations were separated on short gradient SDS-PAGE. Gels were then sliced into two bands per lane, subjected to in gel Trypsin digestion and analyzed by LC-MS/MS. Protein identification from raw data was performed using Max Quant v1.5.5.1 with 1% FDR for both peptides and proteins. Proteins detected in the two replicates were selected to establish the reference PND14 rat forebrain nuclear proteome.

Supplementary Table 2 Enrichment analysis of the PND14 rat forebrain nuclear proteome. Details for enrichment analysis of the nuclear protein dataset against Rattus norvegicus proteome. for GO Cellular Component, GO Molecular Function terms or GO Biological Process terms and REACTOME pathways using the DAVID webtool.

Supplementary Table 3 Nuclear FMRP interactome. Proteins eluted from three independent pull down assays using as GST‐FNT recombinant protein as the bait or GST as the negative control, were separated on gradient SDS‐PAGE, subjected to in gel Trypsin digestion and analyzed by LC‐MS/MS. Protein identification from raw data was performed using MaxQuant v1.5.5.1 with 1% FDR for both peptides and proteins. Proteins with no missing value (MS/MS≠0) in GST‐FNT were selected and used for differential statistical analysis using the SAINTexpress tool. Proteins with a cut off of FC >2 and SP>0.5 were significant and protein with a cut off of FC>3 and SP>0.7 were considered as high confidence interactor.

Supplementary Table 4 Annotation analysis of the nuclear FMRP interacting protein dataset. Details for UniProt Keyword (UP‐KW) annotation analysis of the human homologs of the nuclear FMRP interacting protein dataset using the DAVID webtool for UP‐KW Cellular Component, UP KW Molecular Function, UP‐KW Biological Process terms or UP‐KW Disease.
