# Supplementary material for: Combining affinity purification and mass spectrometry to define the network of the nuclear proteins interacting with the N-terminal region of FMRP
Source: Front Mol Biosci. 2022 Sep 27;9:954087. doi: 10.3389/fmolb.2022.954087 (PMC9553004; doi:10.3389/fmolb.2022.954087)
Supplement: Supplementary file 5 [file DataSheet1.docx]

**Supplementary Material and Methods:**

**SDS-PAGE and Western Blotting**

Samples from the nuclear fractionation or pull down elution samples were resolved by SDS-PAGE, transferred onto nitrocellulose membrane (BioTrace NT, Pall Corporation) and immunoblotted using primary antibodies as indicated in the Supplementary Material. Primary antibodies were detected using the appropriate horseradish peroxidase (HRP)-conjugated secondary antibodies (GE healthcare). Proteins were then visualized using chemiluminescent solutions and images acquired on a Fusion FX7 system (Vilber Lourmat).

**List of antibodies used in western blotting and in PLA**

|  | **Antibodie name** | **Company** | **Species** | **Dilution** |
| --- | --- | --- | --- | --- |
| **WESTERN BLOTTING** | anti-Synaptotagmin | Stressgen, SYA-148 | Mouse | 1 :2000 |
|  | anti-PSD95 | Millipore, AB9708 | Mouse | 1 :10000 |
|  | anti-CoxIV | Abcam, ab14744 | Mouse | 1 :2000 |
|  | anti-Calnexin | Santa Cruz, sc-23954 | Mouse | 1 :500 |
|  | anti-GM130 | BDBiosciences, 610823 | Mouse | 1 :500 |
|  | anti-Nopp140 | Santa Cruz, sc-374033 | Mouse | 1 :700 |
|  | anti-Fibrillarin | Invitrogen,MA3-16771 | Mouse | 1 :2000 |
|  | anti-Histone H4 | Active-motif, 39269 | Rabbit | 1 :2000 |
|  | Anti-FMRP | Martin lab, 2F5 | Mouse | Hybridome 10% serum |
|  | Anti-Poldip3 | Santa cruz,sc-398931 | Mouse | 1 :100 |
|  | Anti-FXR1P | Bardoni lab, 830 | Rabbit | 1 :2000 |
|  | Anti-Ddx41 | Santa Cruz, sc-166225 | Mouse | 1 :100 |
| **PLA** | Anti-FMRP | Abcam, 17722 | Rabbit | 1 :500 |
|  | Anti-FMRP | Bardoni lab, 1C3 | Mouse | 1 :50 |
|  | Anti-FXR1P | Santa Cruz, sc-374148 | Mouse | 1 :50 |
|  | Anti-Ddx41 | ABClonal, wh231979 | Rabbit | 1 :50 |
|  | Anti-Poldip3 | Santa cruz, sc-398931 | Mouse | 1 :50 |
|  | Anti-Hnrnpa3 | Biorbyt, orb324945 | Rabbit | 1:50 |

**Buffers composition**

Hypotonic buffer at 1.5mM MgCl2:

10mM HEPES-KOH pH 7.9

10 mM Tris-HCl pH 7.4

10mM KCl

1.5mM MgCl2

0.5mM DTT

1X complete EDTA-free protease inhibitor cocktail Roche

Hypotonic buffer at 0.5mM MgCl2:

10mM HEPES-KOH pH 7.9

10 mM Tris-HCl pH 7.4

10mM KCl

0.5mM MgCl2

0.5mM DTT

1X complete EDTA-free protease inhibitor cocktail Roche

Native lysis buffer:

50mM Tris Ph7.5

150mM NaCl

5mM MgCl2

1% Igepal

0.5% DOC

complete EDTA-free protease inhibitor cocktail Roche

Wash buffer:

25mM Tris ph7.5

150mM NaCl

1mM DTT

250 μg/ ml Pefabloc

High stringency wash buffer:

25mM Tris ph7.5

500mM NaCl

1mM DTT

250 μg/ ml Pefabloc

**Mass spectrometry analysis**

*Sample preparation:* Protein from samples were stacked by short migration (≈1cm) into a Bis-Tris gradient gel (4–15%, Mini-PROTEAN, BioRad) and colored by Coomassie staining (Imperial blue, Thermo Fisher Scientific). Each lane was manually excised into two bands of ≈0.5cm for the nuclear extracts and into one band of ≈1cm for the pull down samples, which were further cut into 1 mm^3^ cubes. Proteins in gel pieces were reduced by 10 mM dithiothreitol and alkylated with 55 mM iodoacetamide and then digested over night at 37°C with trypsin at 10 ng/µL (PIERCE, Thermo Fisher Scientific) in 50mM ammonium hydrogen carbonate in water. Tryptic peptides were extracted with 1% formic acid and after acetonitrile, dried and resuspended in 0.1% (v/v) formic acid. The resulting peptides mix were then subjected to LCMS/MS analysis.

*NanoHPLC-Q-exactive plus analysis:* Peptide separations were carried out on a nanoHPLC (ultimate 3000, Thermo Fisher Scientific). 5 µL of peptidic solution was injected and concentrated on a µ-Precolumn Cartridge Acclaim PepMap 100 C18 (i.d. 5 mM, 5 µm, 100 Å, Thermo Fisher Scientific) at a flow rate of 10 µL/min and using solvent containing H2O/ACN/FA 98%/2%/0.1%. Next, peptides separation was performed on a 75 µm i.d. x 500 mM (3 µm, 100 Å) Acclaim PepMap 100 C18 column (Thermo Fisher Scientific) at a flow rate of 200 nL/min. Solvent systems were: (A) 100% water, 0.1%FA, (B) 100% acetonitrile, 0.08% FA. The following gradient was used t = 0 min 4% B; t = 3 min 4%B; t = 170 min, 35% B; t = 172 min, and 90% B; t = 180 min 90% B (temperature set at 35◦C). The nanoHPLC was coupled via a nanoelectrospray ionization source to a Hybrid QuadrupoleOrbitrap High Resolution Mass Spectrometer (Thermo Fisher Scientific). MS spectra were acquired at a resolution of 70 000 (200 m/z) with a scan range of 150-1800 m/z, an AGC target value of 5e5 and a maximum injection time of 50 ms. 10 most intense precursor ions were selected and isolated with a window of 2 m/z and fragmented by HCD (Higher energy C-Trap Dissociation) with normalized collision energy (NCE) of 27. MS/MS spectra were acquired in the ion trap at a resolution of 17 500 (200 m/z) with an AGC target value of 2e5 and a maximum injection time of 100 ms.

*Protein identification and quantification:* MS raw data were analysed using using MaxQuant 1.5.5.1. The derived peak list was searched with the built-in Andromeda search engine against the Rattus Norvegicus UniprotKB database (Version Dec2020) using the following search parameters: trypsin digestion with a maximum of two missed cleavages, 7 amino acids for minimum required peptide length, with acetyl (protein N-term) and oxidation (M) set as variable modifications and carbamidomethyl (C) as a fixed modification. As no labeling was performed, multiplicity was set to 1. A protein false discovery rate (FDR) of 0.01 and a peptide FDR of 0.01 were used for identification level cutoffs with a minimum unique peptide of 1. The match between runs option was also enabled with a match time window of 0.7 min and an alignment time window of 20 min. The “proteinGroups” output file from MaxQuant is available in the supplement.

*Protein selection:* Hits to the reverse database, contaminants and proteins only identified with modified peptides were eliminated. To establish the nuclear proteome, proteins present in both nuclear lysates with no missing values in MS/MS count or Intensity were selected. To sort out proteins differentially interacting with GST-FNT *versus* GST, a statistical analysis were performed using the SAINTexpress tool [1] available as part of the web-accessible Reprint-CRAPome resource (<https://reprint-apms.org/>, [2]). Analysis was run using "Other" as Organism, "AP-MS" as Experiment Type and "SPC" as Quantitation type to upload data, the default settings to calculate the empirical fold change (FC) and the SAINTexpress option to calculate the Probabilistic SAINT Score (SP). As previously reported [3], we used peptide counts information (MS/MS counts) as input. Moreover, only proteins with no missing MS/MS count value (MS/MS count ≠0) in GST-FNT triplicates were selected and uploaded. Lastly, as recommended by Guard et al. [3], we applied a cutoff of FC-A > 3 and SP > 0.7 to select “high-confidence” interactors. The “medium confidence” interactors were the remaining preys presenting the minimal cutoffs proposed by the software, *i.e.* FC-A > 2 and SP > 0.5.

[1] SAINTexpress: improvements and additional features in Significance Analysis of INTeractome software. Teo G, Liu G, Zhang J, Nesvizhskii AI, Gingras AC, Choi H. J Proteomics. 2014 Apr 4;100:37-43. doi: 10.1016/j.jprot.2013.10.023. Epub 2013 Oct 26. PMID: 24513533

[2] The CRAPome: a contaminant repository for affinity purification-mass spectrometry data.

Mellacheruvu D, Wright Z, Couzens AL, Lambert JP, St-Denis NA, Li T, Miteva YV, Hauri S, Sardiu ME, Low TY, Halim VA, Bagshaw RD, Hubner NC, Al-Hakim A, Bouchard A, Faubert D, Fermin D, Dunham WH, Goudreault M, Lin ZY, Badillo BG, Pawson T, Durocher D, Coulombe B, Aebersold R, Superti-Furga G, Colinge J, Heck AJ, Choi H, Gstaiger M, Mohammed S, Cristea IM, Bennett KL, Washburn MP, Raught B, Ewing RM, Gingras AC, Nesvizhskii AI. Nat Methods. 2013 Aug;10(8):730-6. doi: 10.1038/nmeth.2557. Epub 2013 Jul 7. PMID: 23921808

[3] Label-Free Immunoprecipitation Mass Spectrometry Workflow for Large-scale Nuclear Interactome Profiling. Guard SE, Ebmeier CC, Old WM. J Vis Exp. 2019 Nov 17;(153). doi: 10.3791/60432. PMID: 31789314

**Supplementary Figures:**

**
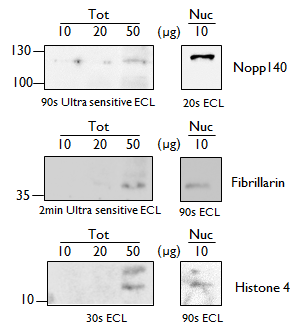
**

**Supplementary Figure 1: Detection of Nopp140, Fibrillarin and Histone H4 in the total lysate from PND14 rat forebrain.** To appreciate the levels of the nuclear markers Nopp140, Fibrillarin and Histone H4 in the total lysate of PND14 rat forebrain compared to the nuclear fraction, 10 µg, 20 µg and 50 µg of proteins from the total lysate (Tot) or 10 µg of proteins from the nuclear fraction (Nuc) were subjected to immunoblotting using the indicated antibodies and then the appropriate horseradish peroxidase (HRP)-conjugated secondary antibodies. Proteins were visualized using the Enhanced chemiluminescence western blot kit from Millipore (ECL) or the SuperSignal West Femto Maximum Sensitivity substrate from Thermo Scientific (Ultra sensitive ECL) at the indicated time of acquisition. Our results indicate that the visualization of the nuclear markers in the total fraction compared to their detection in the nuclear lysate requires higher amount of proteins, associated with longer time of exposure and/or ultra sensitive ECL substrate, thus reflecting a clear enrichment of the nuclear fraction in nuclear proteins.


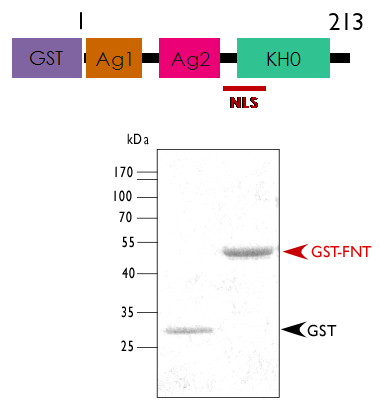


**Supplementary Figure 2: Description of the GST-FNT bait.** High panel: Schematic diagram of the human N-terminal fragment (1-213aa) fused to the GST tag used in this study (GST-FNT). Major domains (Ag1, Ag2, KH0) and the nuclear localization signal (NLS) of FMRP are highlighted. Low panel: Coomassie stained gel of the purified GST and GST-FNT recombinant proteins. Black arrow indicates GST and red arrow indicates GST-FNT.


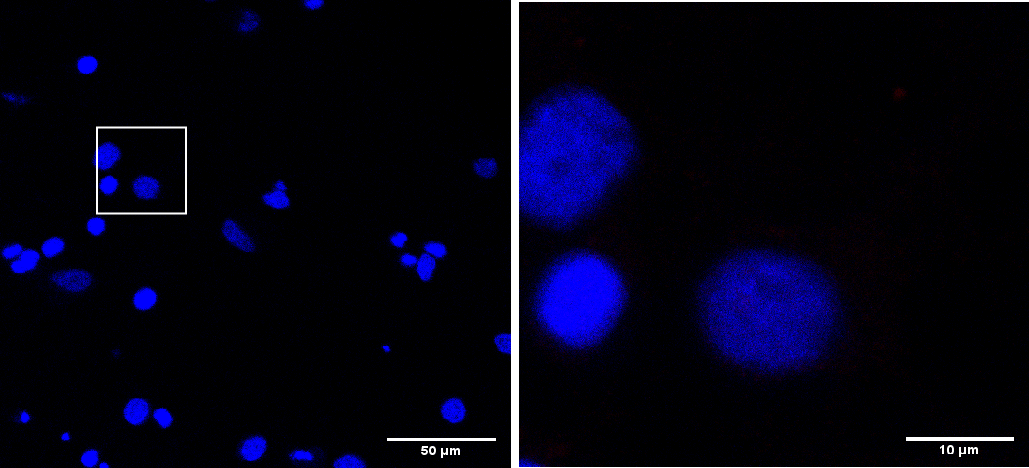


**Supplementary Figure 3: Negative control for Proximity Ligation Assays (PLA).** Representative confocal images of primary hippocampal neurons in culture processed with the PLA Duo-link® kit (Sigma-Aldrich) without primary antibodies (Scale bar = 50 μm). Enlargement of delimited area is also shown (Scale bar = 10 μm). No PLA dots could be detected as background signal.

**Supplementary Tables legend:**

**Supplementary Table 1:** PND14 rat forebrain nuclear proteome.

10 µg of proteins from two independent nuclear preparations were separated on short gradient SDS-PAGE. Gels were then sliced into two bands per lane, subjected to in gel Trypsin digestion and analyzed by LC-MS/MS. Protein identification from raw data was performed using Max Quant v1.5.5.1 with 1% FDR for both peptides and proteins. Proteins detected in the two replicates were selected to establish the reference PND14 rat forebrain nuclear proteome.

**Supplementary Table 2:** Enrichment analysis of the PND14 rat forebrain nuclear proteome.

Details for enrichment analysis of the nuclear protein dataset against Rattus norvegicus proteome. for GO Cellular Component, GO Molecular Function terms or GO Biological Process terms and REACTOME pathways using the DAVID webtool.

**Supplementary Table 3:** Nuclear FMRP interactome.

Proteins eluted from three independent pull down assays using as GST-FNT recombinant protein as the bait or GST as the negative control, were separated on gradient SDS-PAGE, subjected to in gel Trypsin digestion and analyzed by LC-MS/MS. Protein identification from raw data was performed using MaxQuant v1.5.5.1 with 1% FDR for both peptides and proteins. Proteins with no missing value (MS/MS≠0) in GST-FNT were selected and used for differential statistical analysis using the SAINTexpress tool. Proteins with a cut off of FC >2 and SP>0.5 were significant and protein with a cut off of FC>3 and SP>0.7 were considered as high confidence interactor.

**Supplementary Table 4:** Annotation analysis of the nuclear FMRP interacting protein dataset.

Details for UniProt Keyword (UP-KW) annotation analysis of the human homologs of the nuclear FMRP interacting protein dataset using the DAVID webtool for UP-KW Cellular Component, UP KW Molecular Function, UP-KW Biological Process terms or UP-KW Disease.
